# It’s okay to breastfeed in public but…

**DOI:** 10.1186/s13006-019-0216-y

**Published:** 2019-06-11

**Authors:** Athena Sheehan, Karleen Gribble, Virginia Schmied

**Affiliations:** School of Nursing and Midwifery, Western Sydney University, Locked Bag 1797, Penrith, NSW 2751 Australia

**Keywords:** Breastfeeding in public, Sexualisation of the breast, Men and breastfeeding

## Abstract

**Background:**

Decisions about infant feeding are embedded and are continuously made within a woman’s social and cultural context. Despite the benefits of breastfeeding to both women and infants, and government policies and laws to protect and promote breastfeeding, breastfeeding in public remains a controversial issue. The purpose of this paper is to present findings from an Australian study that explored the perceptions and beliefs held by first time expectant mothers and their family and social networks towards breastfeeding in public.

**Methods:**

This study collected data through fifteen family conversations to explore the views and beliefs of first time mothers and those in her social network towards breastfeeding. Breastfeeding in public was discussed in nine of the family conversations with 50 individual people contributing. We used a process of a descriptive contextual analysis drawing out specific elements of the family conversations to identify an underlying ideology around breastfeeding in public within these groups.

**Results:**

The analysis focused on four key elements of the conversations. These included the descriptions of the event, the actions, the locations and feelings of the dominant players. Descriptions of the event outlined different beliefs and feelings related to breastfeeding in public and whether it should occur at all. Suggestions for not breastfeeding in public were timing your outings so feeding could take place at home, expressing breastmilk or using the dummy. When breastfeeding in public was considered acceptable, there were requisite social norms. Breastfeeding in public requires women to be discrete and covered-up, so as not to expose her breast. She is also required to feed in an appropriate place to avoid discomforting others, guard against judgement, and to protect herself from the unwanted male gaze.

**Conclusions:**

Our findings suggest that controversy remains as to whether breastfeeding should occur in public at all. Even where breastfeeding in public is seen as a woman’s choice, there are social rules that govern how it should be undertaken to make it an ‘appropriate’ activity. As a result, women need to take responsibility for others feelings, minimise the discomfort of others and ‘keep themselves safe’ if and when they breastfeed in public spaces.

## Background

Globally, breastfeeding is important as it optimally supports the health and well-being of mothers and infants and is the most ecologically sustainable way to feed an infant [1, 2]. Because of the benefits of breastfeeding, international policies and strategies have been developed to improve breastfeeding initiation and duration rates, including the Baby Friendly Hospital Initiative [3, 4]. Yet breastfeeding initiation and duration rates across the globe vary enormously. In many resource rich countries such as Australia, most women initiate breastfeeding but this drops steadily in the first 2 months. Decisions about infant feeding are embedded and are continuously being made within a woman’s social and cultural context [5, 6]. Research demonstrates that breastfeeding in public remains a controversial issue and embarrassment to breastfeed in public is an influencing factor in women’s infant feeding decisions [7].

Australia boasts high breastfeeding initiation rates with the most recent National Infant Feeding survey reporting that breastfeeding was initiated for 96% of children zero to 2 years of age [8]. However, breastfeeding can be difficult to sustain and early cessation of exclusive and any breastfeeding remains a significant problem in Australia. Only 15% of children are exclusively breastfed to 5 months and only 60% are breastfeeding at all at 6 months of age [8]. This falls well short of the recommendations of the World Health Organization (WHO) and the Australian government [4, 9]. Early cessation of breastfeeding is more common where women are from disadvantaged communities [10] and the difference in breastfeeding rates, compared to those of the most advantaged groups, is widening [11]. This is likely to compound the health disadvantage for these infants and their mothers [8, 11].

One of the challenges faced by women in maintaining breastfeeding is the social stigma associated with breastfeeding in public in Australia. National and state/territory legislation frames breastfeeding as a human right and makes it unlawful for women to be discriminated against because of breastfeeding. However, the belief that women should not breastfeed in public places or should only do so in prescribed ways is commonly held [12]. Social disapproval of breastfeeding in public is long-standing. McIntyre et al. [13] found that 82% of 2000 study respondents agreed that bottle feeding is more acceptable in public than breastfeeding, and 48% agreed that men are bothered by breastfeeding in public. Mainstream media commonly reports on incidents where women have been told that they cannot breastfeed in cafés, shops, galleries, swimming pools or in parliament [14–18]. Such reports are often accompanied by polls asking whether women should breastfeed in public [15]. This undermines the position of breastfeeding as a human right and reinforces the belief amongst many mothers and others that breastfeeding in public is a questionable practice [19, 20].

Social disapproval of public breastfeeding is attributed to the sexualisation of the breast. Breasts have dual functions in Western contexts: as a body part that is sexually attractive and involved in sexual activity but that also provides milk and nurture to children [21]. Although, it is important to note that the sexual function of breasts is culturally determined rather than inherent, the perception of breasts as sex organs impacts upon how women who use their breasts for their milk producing function are perceived and treated [5, 22]. It has been identified that women who breastfeed in public may be accused of sexual exhibitionism or sexual immorality [21, 23]. However, such censure is not about breasts being seen, as breasts are ubiquitous in Western advertising, fashion and media [21, 24]. Rather, it appears that the discomfort with breastfeeding in public is a result of a perceived conflict between women as sexual beings and as mothers [24, 25]. The discomfort of others with breastfeeding transfers onto women who express feeling nervous, anxious, embarrassed, exposed, intimidated and confused about breastfeeding in public [26, 27].

Women manage the stigma associated with breastfeeding in public in a variety of ways. Some avoid breastfeeding in public altogether. UK research from 2010 identified that overall 40% of breastfeeding women had never breastfed in public with those who were young or from socially disadvantaged backgrounds being even less likely to do so [28]. Avoiding breastfeeding in public requires women to restrict their movements or to feed their babies infant formula or expressed breastmilk when away from home. Indeed, even the intent to exclusively breastfeed is greater in women who are comfortable with the idea of breastfeeding in public [29]. Women may also engage in harm minimisation by choosing to breastfeed in locations where breastfeeding is deemed less offensive or by employing tools such as breastfeeding covers [26, 30]. Some find courage to breastfeed in “power by numbers,” that is with peers who are also breastfeeding [26].

While public health advocates prioritise breastfeeding because of its importance to health, as with all health behaviours’, decisions concerning infant feeding are made within a whole of life context [6, 31]. Decisions about infant feeding including breastfeeding in public are however, not just informed by health considerations, rather, they are embedded, and are continuously being made, within a woman’s socio-cultural context, powerfully shaped by class, education, ethnicity and age [11, 32]. High levels of self-efficacy, more likely fostered within an economically advantaged milieu [33], influence breastfeeding success and foster opportunities for the woman to develop a positive nurturing relationship with her baby [34, 35]. Conversely, women who have less social support are less able to seek help with breastfeeding problems [36]. However, regardless of social status and education, the views and actions of a woman’s family and close friends has a powerful impact on her infant feeding decisions [37]. The aim of this study was to explore the perceptions and beliefs held by first time expectant mothers and their family and social networks towards breastfeeding in public, using family conversations.

## Methods

In this paper we present the perceptions and beliefs held by first time expectant mothers and their families and social networks towards breastfeeding in public. Data for this study were drawn from a larger in-depth qualitative study funded by the Australian Research Council that aimed to ‘ascertain the potential impact that family and friends had on the early parenting experience and infant feeding decision-making of first-time mothers’. Data were collected using family conversations with 15 women from an outer metropolitan area of Sydney and their families or close social network participated in this study [38]. Overall there were 89 participants in the larger study.

For this analysis, data was drawn from nine family conversations, as they were the only groups to discuss breastfeeding in public. Family groups ranged from 2 to 7 people (a total of 50 participants), and the conversations took place between 34 and 36 weeks of the woman’s pregnancy. The age range of the mothers was 19–35 years and all were first time mothers. All mothers were born in Australia, apart from one woman born in the Pacific Islands. Seven of the woman held tertiary degrees, one had completed a TAFE (Technical and Further Education) course and one had completed year 12 schooling. While the outer metropolitan area of Sydney where this study was conducted has areas of varying levels of disadvantage/advantage, it has a low overall SEIFA (Socio-Economic Indexes for Areas) Index making it an area of socioeconomic disadvantage when compared to other areas nationally [39]. When asked about their financial situation eight of the women stated they were financially comfortable or fairly comfortable and one women stated her financial situation was extremely difficult. The groups included the pregnant woman, her partner, and a mix of sisters, the woman’s mother (MGM), mother in law (PGM), the woman’s father (MGF), father in law (PGF), aunties and friends. The family conversations were audio-recorded and later transcribed. An earlier paper describes the family conversations as a method of data collection in more depth [38]. All names in the data have been changed to protect the participants’ confidentiality.

Data for this component of the study was analysed using a descriptive analysis of context. Halliday [40] (p.12), talks about three features of context, the field, the tenor and the mode. These three features, broadly speaking reveal; what is happening (the field), who is taking part (the tenor), and what is being achieved (mode). In order to disclose these contextual features, we chose to examine a number of key elements of the discourse relating to the construction of breastfeeding in public. The chosen elements included: descriptions of the event of breastfeeding in public, descriptions of the mother, actions and locations relating to breastfeeding in public, and feelings about breastfeeding in public. Some of these elements were then examined more closely. For actions we specifically looked at the ‘mothers’ actions’, ‘the actions of others’ and for feelings, the ‘feelings of the mother’ and the ‘feelings of others’. Key concepts were then identified within each of these elements. Finally, the concepts were drawn together to identify an overarching dominant ideology. For the purposes of this analysis, we refer to Hasan’s definition of ideology ‘as a socially constructed system of ideas’ [41] (p. 256).

## Results

The results are presented using the elements that were examined in the data as main headings. These include ‘descriptions of breastfeeding in public’, ‘descriptions of the mother’s feelings’, ‘descriptions of the observer’s feelings’, ‘the mother’s actions’, ‘the actions of others’ and ‘locations’. Under each of these headings, sub-headings are used to list the key concepts identified within each of these elements. These elements were complimentary to each other and moving between these elements and identifying key concepts within and across these elements, a dominant ideology around breastfeeding in public was seen.

Extracts from the conversations have been used to illustrate the concepts and the underlying ideology. Where a conversation thread is used, the participants place in the family conversation has been identified. For example, the pregnant woman is referred to as the mother and her partner as the father. Other participants are identified by the acronyms listed against the participants in the methods section above. For example, the maternal grandmother is identified by the acronym MGM.

### Descriptions of breastfeeding in public

#### Beliefs around breastfeeding in public

One of the key elements extracted from the family conversations was the description of breastfeeding as an activity in public**.** Descriptions of the ‘activity’ illustrated contrasting beliefs amongst the participants around whether breastfeeding should occur in public at all. Comments ranged from *‘yeah I think it’s accepted today… I don’t even give it a second thought when I see someone breastfeeding in public, I think it’s beautiful, I think its fine’* (Fam 9 MGM) to ‘*I think the majority of people are ok with it, you’ll always have a minority’* (Fam 11 MGM) and then finally, ‘*no, it’s (breastfeeding) not (accepted in public)* (Mother Fam10).

*Mother: No, it’s (breastfeeding) not (accepted in public). But in xxx (their country of birth) they do cause they don’t care.*

*Facilitator: They don’t care?*

*Mother: Yeah.*

*Facilitator: But for you being in Australia, they don’t like you to do that?*

*Mother: Yeah. Plus I won’t like that.*

*Facilitator: You wouldn’t like it.*

*Father: Breastfeeding’s a personal thing. (Family 10)*

As seen in the above conversation and the one below, there were comments in the descriptions around breastfeeding in public that highlighted that breastfeeding for some was a private and personal matter.

*Mother: Maybe I’ll feel different once I have her but I feel a bit funny about doing it in front of other people. I think I’d rather sit quietly with her and have that as a private moment. Maybe I’ll feel different when I have her and she’s hungry and I just need to do it.*

*MGM: I think the breastfeeding is a time you spend with your baby anyway. It’s not a social time, I guess maybe if Beth was here with her friends, like Ashleigh and Tara who have had babies, I can see its fine to sit out and feed then. But if you’ve got people over and a lot of them are not close friends.* (Fam 4)

Later in this family conversation the MGM equated breastfeeding to going to the toilet, taking the inappropriateness of breastfeeding in public and the requirement for privacy to a whole new level.

*‘There are certain things and I know it’s a natural function, but there are certain natural functions you don’t need to do in public. You don’t go to the toilet in public and that’s a natural function you know’* (Fam 4 MGM) *‘*

Being a private and personal matter, meant that for some of the participants both men and women, breastfeeding was not an activity that should be undertaken in front of men. This was because it was unapproved of by men or made men feel uncomfortable. Exemplars of these comments included *‘I felt they (older men) didn’t approve of women breastfeeding in front of them*’ (Fam 2 MGM) and ‘*some men don’t like it…’* (MGM Fam 1).

#### Managing her behaviour

Even when the participants considered that breastfeeding in a public place was ‘acceptable’, there were a number of statements used to describe how the mother should be when feeding in public. These descriptors included being ‘considerate’, ‘discreet’ and ‘safe’. For example, participants made the following comments ‘*as long as you’re considerate when you’re breastfeeding it shouldn’t matter’* (Fam 11 Mother), ‘*there are always ways women can do it discretely’* (Fam 4 MGM), and ‘*you’ve just got to keep yourself safe as well you know’* (Fam 11 MGF). The concept of being safe is raised further in this paper.

### Description of the mothers feelings

Descriptions of the mother’s feelings as well as what others considered the mother felt when breastfeeding in public were evident in the recorded conversations**.** For the women themselves they described a variety of personal feelings about breastfeeding in public ranging from ‘it’s my right’, ‘not being too concerned’ through to ‘being embarrassed’ and ‘stressed’.

*I don’t think I’ll be too concerned about other people, I’m not prudishly worried and if they’re going to be here that’s tough titties (laughing). I think if they’re here I’m going to do what I feel comfortable with and I don’t think I’m really going to stress about making anyone else uncomfortable. And there’s not really anyone that will be around at the time that I think I will be uncomfortable with (Mother Fam 8).*

This is an interesting quote because while the mother states she won’t be concerned about breastfeeding in front of others she does recognise the potential for discomfort both for herself and others.

In the following quote a friend describes how trying to cover up rather than feed in front of others made her stressed.

*‘but when you’re trying to breastfeed, people are wanting to come and see the baby and you’re not feeling that comfortable and you’re stressed out, it makes it harder. I reckon that’s why I got mastitis because I was stressed about trying to cover myself up when I was feeding and I think I wasn’t feeding properly (Friend, Fam 8).*

For the following woman she was clear that she would not feel comfortable breastfeeding in public at all.

*‘I won’t like that (breastfeeding in public) (Mother Fam10).*

Descriptions of how others considered the mothers were feeling when breastfeeding in public were also voiced in the conversations. Participants in the groups generally believed that if a woman breastfed in public she must feel comfortable about it or indifferent about how other people in the public might feel.

*Father: How does she feel, it’s her boob being exposed, how do you think she feels about it.*

*MGM: She’s comfortable if she’s sitting there in a public place doing it. (Fam 11)*

*and*

*Aunt: You do get people (breastfeeding women) that have attitudes and they don’t care. They really don’t care (Fam 1).*

### Description of the observers feelings

Feelings of others in relation breastfeeding in public ranged from feeling embarrassed to feeling fine but with a proviso that the woman was not exposed. *‘I’m fine, as long as the exposure of it all is not…*(Aunt Fam 1),

and

*Mother: I think it should be covered up, not necessarily hidden away if there is nowhere to go, like if you’re, I don’t know, on a, not a bus, but somewhere where you can’t go to the bathroom or a parent room, you should have something that you are able to cover, like you know what I mean? Like that*

*Facilitator: Some way to shield everyone else from seeing*

*Mother: Baby sling that you can kind of turnover on that side, so the baby just sees what it needs to get to, what it needs to get to without exposing it to everybody and making everyone feel uncomfortable. (Fam11)*

In particular, and seen in earlier excerpts of conversation, was concern regarding men’s feelings when women were breastfeeding in public. These concerns were highlighted by both women and men in the conversations with comments such as *‘from a male perspective I feel uncomfortable when someone is breastfeeding and I’m there (*Father Fam 2*)* and ‘*some men don’t like it…’* (MGM Fam 1) and *my other son gets embarrassed* (PGM Fam 4).’

These findings correlate with other findings in this study that women need to be discrete and covered when breastfeeding in public and arguably that the woman needs to be mindful of the comfort of others.

### The Mother’s actions

The data revealed a number of actions of both the mother and others in relation to the practice of breastfeeding in public. When the actions of the mother, were collated, what became obvious was that there were both appropriate and inappropriate actions in terms of breastfeeding in public.

#### ‘Appropriate’ actions of the mothers

A number of appropriate actions for a mother to breastfeed in public were raised in the conversations. These actions included covering up and putting it away.

##### Covering up

The action of ‘covering up’ was the most common appropriate action identified in the data and was expressed by both women and other participants in the study. For example, I *just think I’ll use a muslin wrap for that extra bit of privacy if I am sitting in a public space* (Mother Fam 2)’; *‘I covered myself’* (PGM Fam 11) and ‘*You have to cover yourself, for me personally I wouldn’t expose myself’ (*MGM Fam 9). Notice that the last quote is in fact identifying two actions (covering and exposing) which juxtaposes what is considered an appropriate action with an inappropriate action. This usage suggests that if a woman does not cover up when breastfeeding in public, she is as good as baring herself.

Other participants such as the grandparents, partners and friends also spoke about the need for women to cover up stating, ‘*I’d rather her to be covered up’* (Father, Fam 4), and ‘*I do like a lady to cover up a little bit’* (Aunt Fam1). The following extract again contrasts an appropriate action ‘cover yourself ‘with an inappropriate action ‘show yourself’. Further to this, the activity of ‘showing yourself’ is described as ridiculous.

*The cover, cover yourself. I never breastfed in a room full of people, maybe because where we came from it was always respect, men especially when around, but never women to breastfeed in front, this is in xxx I’m talking about, of the society or men or friends or whatever. Always private or cover properly like if you’re in places you can’t go somewhere else, you just cover yourself, you can’t just take your breast out. It’s ridiculous I think to do that in public in shopping centres, or things like that. (MGM, Fam2).*

Again the word usage ‘take your breast out’ suggests that the woman is excessively exposing herself.

##### Putting it away

A key concept deemed as an appropriate action for the mother, was ‘putting your boob away’ (Fam 11). Putting the boob away could negate the need to breastfeed in public. Actions that supported putting the boob away included timing the feed, expressing milk, and giving a dummy.

*But if you time it well too, what you do is your feed is the last thing you do before you leave the house. Pack everything up, get ready and sort of time it. You can work it out usually that you feed the baby, then you leave the house. You might not even have to do it before you come home, if you don’t go for an excessive amount of time. You might not have to worry about it. Yeah so. The dummy – that’s where sometimes the dummy comes in handy. (Aunt Fam 1)*

*Mother: That’s like me, if I had to go out, I’d express milk before I’d go (Fam11)*

*and*

*Mother: Oh just go home and feed the baby there.*

*Facilitator: Oh, ok. So you would never do it in a public place.*

*Mother: No*

*Facilitator: Even if you cover up?*

*Mother: If not, I’ll just try to squeeze it into a bottle.*

*Facilitator: Oh, squeeze it out. So, express.*

*Mother: Yeah.*

*Facilitator: And then you can go out.*

*Mother: Yes. (Fam 10)*

#### Inappropriate actions of the mother

Again both the women and others made comments that identified inappropriate actions of the breastfeeding woman. Inappropriate actions included getting your boobs out (a euphemism for exposing your breasts) and showing off.

##### Getting your boobs out

Contrasting with putting your boobs away and reinforcing the sense that a woman needs to be discrete and cover her breasts when breastfeeding in public, were comments that highlighted it was ‘inappropriate’ to just get your boobs out.

‘You can’t just take your breast out’ (MGM Fam 2).

This next excerpt highlights the need to be discrete and again emphasises the woman’s responsibility for others comfort; because ‘some men don’t like it’ and ‘it’s not for everybody’.

MGF: There’s women out there that just hang it out and you know.

Aunty: They don’t care.

MGM: Some men don’t like it…

MGF: They just need to be discreet about it. That’s all. It’s not for everybody.

Aunty: It’s not.

Sister: I see so many women out there who manage to do it, without showing anything to anybody. It’s like well, you know, there’s no problem with that. (Fam 1)

##### Showing off

Not only was there concern that a woman would be exposing herself if she breastfed in public, there was a sense that breastfeeding in public could also be seen as a form of exhibitionism. The accusation that a woman was showing off, appeared to occur if she was not suitably discrete or was seen breastfeeding in an inappropriate location.

MGM: *Even if you have to in public there are always ways women can do it discretely without everyone having to see. It’s not something you’re there to show off*.

Later the MGM expanded on the above comment saying…..

‘*I think especially some of the things they’ve had in the papers and on the news about the politician that wanted to feed her baby in parliament and things like that, I don’t think she needed to make that statement, there was no need for it, she didn’t need to take the baby in and feed it’.* (Family 4).

#### The actions of others

As well as looking at the actions of the mothers, the text was also examined for the actions of others in relation to breastfeeding in public. The actions by others included perving, commenting, and judging.

#### Perving

One of the actions identified in the data was that of ‘perving’. Men were accused of perving *‘he wanted a perv’* (MGF Fam 4). In one case while a man admitted to ‘looking’ suggesting this was a normal male behaviour, he objected to the accusation that he was a pervert ‘*you know, like, of course I had a bit of a look you know, and like I’m normal but then her partner came and sat down, and I heard her say, this perverts looking at my boobs’* (Male: Fam 11).

Perving is derived from the word perv which can be defined as a lustful or lecherous look. In the second quote the speaker admits to having a ‘look’ and arguably a lustful look, as he then suggests a shared assumption that normal (presumably heterosexual) men would do this and therefore he is not a pervert which is defined as a person whose sexual behaviour is regarded as unacceptable. This reinforces the view that the breast is consistently viewed as sexual even when performing a functional role.

Given the sexualisation of the breast, and the potential for being accused of perving, it was not surprising then that for some men, even when they acknowledged it was the woman’s right to breastfeed in public they felt uncomfortable.

*I don’t think they should be banished to the shadows to do it as I realise it’s my problem not theirs and they’re entitled to do it and I’m not saying they shouldn’t. ‘But it is uncomfortable, particularly when they are talking to you and you don’t know where to look (laughing) and pretend it’s not happening. I guess it is uncomfortable for a male because you just don’t know what protocol is but certainly I’m not from the point of view that they’re the problem, it’s me that’s the problem* (Father Fam 2).

#### Protecting

While not an action of others but rather a requirement of the mother, the view that a breastfeeding woman could be ‘perved on’ appeared to be the basis for the woman to keep safe and protect herself ‘*You’ve just got to keep yourself safe as well you know (MGF Fam 11).*

As the following quote suggests, the concept of being safe was connected to the concern regarding how people would react in the public.

*Mother’s sister: Yeah I definitely don’t like that idea I was [unclear] the other day, a lady was sitting there in the middle of the shops, in the middle of the aisle, breastfeeding and her boobs were out. But the thing is, [unclear] some people will be rude about it, like “nice tits” whatever, it’ll make them feel uncomfortable, I was thinking I would never do that, like I’d never go out in public in a shop and do it, because you never know what, like, you know, not good people are out there.*

*PGM: But yeah, out in public...*

*Mother: You never know what people are thinking or what people are saying you know (Fam 11)*

Later in this conversation the sexual role of the breast emerged suggesting that keeping safe was related to the unwanted sexual gaze.

*MGF: You’ve just got to keep yourself safe as well you know.*

*Father: She’ll be safe.*

*PGM: I think most people don’t want to see it anyhow, they’ll look and then they’ll look away. I don’t think they’re going to stare, I don’t think it means anything sexual, it may just mean they haven’t seen that before (Fam 11)*

#### Commenting and judging

Other actions identified in the data demonstrated that women who breastfeed in public make themselves subject to comments and being judged.

*‘People comment and I saw all the men sitting there, like behind her watching and you know, making comments and stuff’* (Mother Fam 4) and *‘people judging you* (Father Fam 4)’.

Extrapolating from the excerpts of conversation used throughout this paper that suggest women who breastfeed in public can be seen as exhibitionists, exposing themselves and ridiculous would demonstrate that they are indeed judged.

### Locations

Another key feature that was found in the data was that location played a significant role in breastfeeding in public. There were both subtle and overt comments within the conversations that identified there were both appropriate and inappropriate locations for breastfeeding to occur. For example, in the following quote while the woman advocates that it is her right to breastfeed in public she also identifies that she would only feed in an appropriate place.

*Mother: No I’m not, I think its natural, I think it’s my right to feed my baby. You can stand there and do the big dance and tell me that it’s inappropriate and I would still do it. I’m not at all phased by other people’s opinion on it, but I’ve made a decision and obviously I’ll try and look at and not find the most public spot and go here we go, but at the same time if it’s an appropriate location and it needs to occur then it will happen. (Fam 5)*

In examining the data, a number of locations were identified and included what was obviously appropriate and inappropriate locations breastfeed.

#### Appropriate locations

Locations that were deemed as acceptable for women to breastfeed included; the house, the bedroom, parent rooms, the women’s section of a store, somewhere quiet, the car. Comments made regarding these appropriate locations included:

*‘I think the first place that you would go is the women’s section of any sort of store because the women understand’* (Father Fam 11), *‘it’s much nicer to go somewhere nice and quiet at least you can get peace* (MGM Fam 4)’ and ‘*Oh just go home and feed the baby there (Mother Fam 10)’.*

#### Inappropriate locations

Locations that were deemed as unacceptable for women to breastfeed included, in public, in a shop, parliament, a bus, hiding away in a corner, in a food court, the toilet, and restaurants. These comments included *‘like I’d never go out in public in a shop and do it’* (Mother Fam 11) *‘taking the baby to a toilet to breastfeed, you wouldn’t eat there yourself* (MGM Fam 9)’.

While the following comment was made by a maternal grandfather who stated it wouldn’t worry him if he saw a woman breastfeeding in public, he highlighted that breastfeeding in a restaurant could be problematic.

*But like sitting in a restaurant or something like that, may be a little bit unnerving because I think some restaurateurs can be a bit disturbed if someone is breastfeeding out in public. Your sitting down having a nice meal and you can be put off. But it really hasn’t worried me that much (MGM Fam 4).*

## Discussion

It has been stated that ‘all conversation is revealing about the shared assumptions of a community, precisely because of its unselfconscious casual nature which marks its deeper social purposes and gives it the air of an activity that is directed toward nothing but the achievement of talk itself’ [41] (p. 258). For this study we explored the perceptions and experiences around breastfeeding in public, using data drawn from nine family conversations.

One of the perceived limitations of this research is that it reports on only nine family group conversations around breastfeeding in public. As such, the findings may not be transferable to other communities in Australia. The groups however, do represent 50 individuals and the findings resonate with reports of the experiences and reactions of other people and groups regarding breastfeeding in public in Australia [14–18, 42]. The findings therefore, contribute to the evidence on the perceptions and beliefs around breastfeeding in public in Australia.

The analysis focused on four key elements of the conversations. These included the descriptions of the event, the actions, the locations, and feelings of the dominant players. These key elements of the conversations and the emergent concepts within these elements, allowed an exploration of the dominant ideology around breastfeeding in public within these groups. While it might be argued that there are other perspectives on breastfeeding in public, in this context, breastfeeding in public was viewed as a contested activity that had social boundaries and rules. The key findings from this research demonstrated that while there was some controversy over whether breastfeeding in public should occur at all, the main focus of the conversations centred on how breastfeeding in public should be conducted and where.

Comparatively, there were only a small number of participants in this study who considered breastfeeding was not an acceptable activity to occur in public. The acceptability of breastfeeding in public appears to be an ongoing controversy and a number of authors make reference to this. What was clearly evident in our research and similar to other researchers in this field, was that even when breastfeeding in public was seen as acceptable it must be undertaken within a highly regulated and socially acceptable norm [12, 43]. In our research, this highly regulated environment was seen to be one where a breastfeeding woman was discrete, covering herself, and feeding in a location that was deemed appropriate. The sense that a woman should breastfeed ‘discreetly’ is one that is perpetuated in the media and in other social spaces. In 2013, David Koch a television presenter on a breakfast show in Australia created debate with the following statement:

*Women should be able to breastfeed in public...But I don’t think it’s unreasonable to expect breastfeeding in public is done discreetly. I think that’s just a common courtesy to others*. [42]

Similar to our research findings, the debate did not focus on whether women should breastfeed in public but rather, how it should be done. Koch and his supporters, both women and men, urged women to be ‘classy’, ‘discreet’ and ‘courteous’ as they ‘perform’ breastfeeding in public or, as described by cultural theorist Sarah Ahmed, not to be a ‘kill joy’ [44].

While this incident took place in 2013, women in Australia continue to be faced with social disapproval in relation to breastfeeding in public. More recently in 2017 a woman was asked to cover up or feed in a parent’s room by gallery staff as she breastfed her baby in the National Gallery of Australia [18]. Despite a later apology by the Gallery director, the incident exemplifies the continued and vexed ideology around breastfeeding in public and demonstrates the correlation between our findings and those of the general public in Australia. In this discourse, breastfeeding is presented as shameful, to be hidden, and women are required to take responsibility for managing the discomfort of others when breastfeeding in public spaces.

In a study of new migrants in Australia, the perceived lack of visibility of breastfeeding in public led the women to believe that breastfeeding in public was indeed a shameful and inappropriate act [45]. Those findings are congruent with much earlier studies on migrant women from Vietnam [46] who also identified a lack of visibility of breastfeeding in public and the negative impact of this on their infant feeding decisions. Boyer [12] argues that the unease in breastfeeding in public is a significant factor in breastfeeding cessation.

Some could argue that changes have been seen in the acceptability of breastfeeding in public, with Larisa Waters an elected senator for the Australian Greens, breastfeeding her baby in the Australian Senate in early 2017 [47]. The fact that this was so applauded and made headlines when previously breastfeeding senators were asked to leave parliament, further confirms the general overarching invisibility and unease that is felt about breastfeeding in public in Australia. Yet despite the invisibility of breastfeeding in public, comments in our data stating that some women just hang their breasts out or expose themselves suggests excessive baring of the breast. Women could also be perceived as ‘showing off’ if they breastfed in public and were not suitably discrete or breastfed in an inappropriate place. That women are accused of and criticised for deliberately exposing their breasts excessively in public and or breastfeed to make a point has been noted by others [21, 23].

Another key finding in our research was that breastfeeding in front of men was problematic. It wasn’t just the men who raised this issue, women themselves stated it made men feel embarrassed and uncomfortable and there appeared to be a sympathy for this and an implication that it was the breastfeeding woman’s responsibility to put the man at ease. This supports Boyer’s [12] premise ‘that while breastfeeding is meant to occur it is also meant to be hidden in order not to discomfit others, such that undertaking this activity in public can be seen as a deterritorialization of received forms of gendered bodily comportment’ (p. 34). Ahmed’s [44] work on the killjoy suggest that a loss of comfort occurs when others are made uncomfortable by behaviour that is seen as materially different to their own. In this case women breastfeeding in public are seen as killjoys because they are refusing to breastfeed in the prescribed place, or under the required circumstances.

Our research also identified the clash between the breast as a sexual object and as a functional organ. That the breast is highly sexualised in particular cultures has been identified by a number of researchers and commentators [23, 25]. Comments in this research around not breastfeeding in front of men, feelings of awkwardness identified by men, the notion that a man is perving if he looks at a breastfeeding woman’s breast, as well as the comments that suggest a woman needs to be safe, are reflective of a society that is enculturated to see the breast as purely sexual. Mathews [43] has argued that ‘the binary between sexualised erotic breast and functional feeding breast places a great deal of tension on the breastfeeding body’ (p. 14).

That a woman needs to be safe aligns with findings from Henderson et al’s [23] study that found there is fear for women breastfeeding in public of ‘possible predatory attention’. Certainly men were accused of perving in this current research. There were also men however, who recognised that while it was a woman’s right to breastfeed in public they didn’t know the ‘protocol’ for viewing the functional breast. This is not surprising given that men are socialised from an early age to view women’s breasts as sexual only [23]. Our work has perhaps demonstrated that at least for some men this dichotomy between the sexual and functional breast causes tension that they see as their ‘problem’ but do not know how to navigate. This warrants further research to explore ways in which this can be done, because ‘the most important attribute for the maintenance of ideology appears to be its socially constructed inevitability’ [41] (p. 256). While this socially constructed behaviour and belief remains, it can be argued that women will continue to be responsible for managing the comfort of others if they breastfeed in public.

Finally, while the discomfort with breastfeeding in public has been correlated with the sexual focus of the breast, it has also been shown that breastmilk itself can be perceived as a bodily fluid and as such dirty [7, 21, 48]. In our study one of the participants compared breastfeeding in public with going to the toilet. This comment is perhaps suggestive that for this person the correlation between dirty bodily fluids and breastmilk exists.

## Conclusions

Although this data represents only nine family conversation groups, the results are similar to other studies that breastfeeding in public continues to be vexed. The family conversations identified that even when breastfeeding in public is considered acceptable, there are limitations and acceptable social norms that dictate how and when breastfeeding in public should take place. Breastfeeding in public requires women to be discrete. This means women are required to cover up, feed in an appropriate place to avoid discomforting others, guard against judgement, and to protect themselves from the unwanted male gaze.

## Data Availability

The datasets generated and/or analysed during the current study are not publicly available due to confidentiality but are available from the corresponding author on reasonable request.

## Abbreviations

MGF: Maternal grandfather (the pregnant woman’s father)
MGM: Maternal grandmother (the pregnant woman’s mother)
PGF: Paternal grandfather (the pregnant woman’s father-in-law)
PGM: Paternal grandmother (the pregnant woman’s mother-in-law)
